# Ethical considerations of wastewater-based disease surveillance—a qualitative interview study

**DOI:** 10.3389/fpubh.2026.1860050

**Published:** 2026-07-07

**Authors:** James W. Keck, Caroline A. Buchanan, Savannah Tucker, Marla Wehrli, Nancy Kass

**Affiliations:** 1 Institute of Circumpolar Health Studies, University of Alaska Anchorage, Anchorage, AK, United States; 2 Program for Bioethics, University of Kentucky College of Medicine, Lexington, KY, United States; 3 College of Public Health, University of Kentucky, Lexington, KY, United States; 4 Department of Research Services, Alaska Native Tribal Health Consortium, Anchorage, AK, United States; 5 Department of Health Policy and Management, Bloomberg School of Public Health, and Berman Institute of Bioethics, Johns Hopkins University, Baltimore, MD, United States

**Keywords:** community, ethics, public health, qualitative, wastewater surveillance

## Abstract

**Introduction:**

Public health implementation of wastewater-based disease surveillance (WBDS) increased dramatically during the COVID-19 pandemic. WBDS raises unique ethical considerations because of its community-scale method of disease surveillance. However, the perspectives of community members regarding the ethics of WBDS are absent from the literature.

**Methods:**

We explored ethical considerations of WBDS by interviewing community members and professionals across the United States using vignettes and a semi-structured interview process. Vignettes highlighted ethical opportunities and concerns noted in the literature, and interview questions solicited information relevant to Kass’s Public Health Ethics Framework. Professional interviewees were identified from their contributions to the field of WBDS, and community interviewees were recruited through a national survey panel firm. We recorded, transcribed, and qualitatively analyzed interview data using a blended inductive and deductive approach.

**Results:**

We interviewed 8 community members and 13 professionals from February to November of 2023. Interviewees broadly supported WBDS and collectively identified a variety of conditions needed for the ethically acceptable conduct of WBDS, such as transparency, effective communication, data privacy, legitimate purpose, responsible data use, public oversight, equity, and community engagement. Ethical concerns and suggested protective measures were similar between the two interviewee groups with few notable differences.

**Discussion:**

This exploratory study found interviewees were receptive to WBDS when accompanied by transparency, safeguards against data misuse, and clear public health purpose.

## 1 Introduction

Wastewater provides a glimpse into the health of a community. We excrete multiple biomarkers, from infectious pathogens to drug metabolites to stress hormones, all detectable in wastewater. Wastewater-based disease surveillance (WBDS) analyzes these biomarkers to make inferences about the health of communities. WBDS has distinct advantages as a health surveillance tool. It provides information on the health of a population via a single sample, making it efficient and cost effective compared to individual testing (1), and it collects information without collecting individual identifiers.

The COVID-19 pandemic ignited interest in WBDS to monitor SARS-CoV-2 (2). Pandemic-related expansion of wastewater surveillance has encouraged scientists and public health agencies to further advance wastewater science. Next generation sequencing technology enables analysis of wastewater for the presence of all known pathogens (3), including SARS-CoV-2 variants of concern (4). Though most WBDS efforts are focused on infectious disease surveillance, other uses exist, including the monitoring of licit and illicit drug use in communities (5). Scientists have used wastewater to study illicit drug consumption in settings from rural truck stops (6) to college football games (7) to European cities (8).

Wastewater surveillance differs from traditional public health disease surveillance in several ways. First, it measures disease signals within a population sample and not from an individual within the population. Individuals living in communities with wastewater surveillance have not provided explicit consent for analysis of their waste, whereas they would have provided consent for drug or disease testing as an individual. Consent for wastewater testing is impractical because wastewater contains a composite of hundreds to millions of individual samples. Further, communities may not be explicitly informed that community level testing is occurring. While the risk of individual identification from wastewater analysis is negligible, as the number of individuals contributing to a sewershed decreases (e.g., wastewater surveillance of a public housing block), there are increasing risks that individuals living in the surveilled area will be associated (accurately or inaccurately) with the condition being measured. This could lead to stigma (e.g., “students living in that university dormitory use a lot of cocaine”) or public health action impinging on autonomy (e.g., drug testing of students living in the dormitory). These attributes necessitate careful consideration of the ethics of WBDS.

Complicating the ethics of wastewater surveillance are the multitude of stakeholders involved in collecting and acting on surveillance data. Utility companies, municipal and state agencies, private and public (state) laboratories, universities, and private contractors may be involved in wastewater collection and analysis. Access to sewer systems and wastewater samples is often controlled by municipalities or entities that manage the facilities under surveillance. Public health decision-making based on wastewater surveillance data may also include multiple actors. Disclosure of wastewater surveillance activities and results are at the discretion of the agencies and organizations involved; these organizations also have the potential authority to create and enforce ethical guidelines for wastewater surveillance (9). As wastewater surveillance moves beyond the COVID-19 pandemic additional questions about the responsible data use will arise.

A growing body of research has examined ethical considerations of WBDS, particularly since 2020 (10). Most of this published literature is based on expert opinion or analysis of case studies leading to frameworks and guidelines proposals [see, for example, Hrudey et al. (11), Maal-Bared et al. (12), and Bowes et al. (13)]. Three empiric studies were identified in a recently published scoping review (10): two survey studies focused on public awareness and opinions of WBDS, and one interview study that sought the opinions of incarcerated individuals on the use of WBDS for COVID-19 control (14–16). While these bioethical treatments of WBDS raise salient issues, they fail to appreciate the potential breadth of WBDS applications and lack the empiric, contextual input from those involved directly in surveillance activities. To address this knowledge gap, we conducted an empirical interview study to understand professional and community perceptions of ethics issues relevant to WBDS for COVID-19 and other public health issues, including using WBDS to monitor for illicit drug use.

## 2 Methods

### 2.1 Study design, participants, and ethics approval

We conducted an exploratory qualitative interview study to gather perceptions related to the ethics of WBDS. We interviewed individuals from two groups: (1) Professionals, defined as those with professional connections to WBDS and/or the ethics of WBDS; and (2) Community members, defined as individuals living where WBDS was or could be implemented. These two groups were not mutually exclusive in that the professionals were also community members; however, for the purpose of this study their responses were coded as professional. The study received institutional review board (IRB) approval from the Alaska Area IRB (2022–04-016). The participants provided verbal informed consent to participate in this study.

### 2.2 Interview guide

We created a semi-structured interview guide that used a hypothetical news story vignette about WBDS. The vignette described wastewater surveillance for COVID-19 as happening in the interviewee’s community. Throughout the interview twists to the vignette presented additional WBDS scenarios, such as surveilling illicit drug use, geographic scale, consent, data ownership, and surveillance among vulnerable populations. The vignette, twists, and interview probes are included in the Supplementary material. The list of topics explored by the interview emerged from literature review and the investigators’ knowledge of contemporary ethical concerns related to WBDS. We also included specific, open-ended questions meant to garner feedback relevant to Nancy Kass’s Public Health Ethics Framework, a widely used tool for assessing ethical use of public health programs and interventions (17). Following each vignette were interview questions and probes regarding how constructs such as fairness, stigma, privacy, and undue burdens might apply to the vignette. A copy of the interview guide is included in the Supplementary material.

### 2.3 Participant recruitment

Potential professional interviewees were identified by their contributions to literature and reports about WBDS ethical concerns. Investigators purposively selected individuals to include a breadth of technical expertise and perspectives. Snowball sampling identified additional potential interviewees. The principal investigator (JWK) sent a templated recruitment email to potential professional interviewees.

To recruit a diverse group of community interviewees, we engaged a survey panel firm, Qualtrics XM (Provo, UT, USA), with national reach. Qualtrics XM sent survey invitations to individuals randomly selected from their national market research panels. Survey invitations were limited to English-speaking adults 18 years of age or older. The survey included a preamble that briefly described the interview topic (testing wastewater to track community health) and eight sociodemographic questions. Respondents interested in participating completed sociodemographic questions and provided their email address. We received 500 completed survey responses from individuals who provided an email address and expressed interest in participating in an interview. From these respondents the study team created a stratified random sample using self-reported political ideology, educational attainment, and urban–rural dwelling categorical responses. The randomly selected individuals were invited by email to participate in the interview. We used iterative replacement for non-responders and declinations to maintain a sample balanced on the sociodemographic criteria of interest. We cumulatively invited 90 of the 500 survey respondents to participate in a video interview. Of those 90 individuals sent an invitation, 12 individuals responded with interest in participating and 8 individuals participated in an interview. The remaining 4 individuals did not respond to subsequent scheduling emails. We concluded participant recruitment after 21 professional and community interviews because the study proposal and budget had planned for 20 interviews. Given the exploratory nature of this study, we did not use thematic saturation to determine sample size.

### 2.4 Interview procedures

All interviews were conducted virtually and recorded using video conferencing software. An electronic copy of the informed consent form was sent by email to interviewees prior to the scheduled interview. Interviewees provided verbal informed consent and permission for audio recording prior to the start of the interview. JWK conducted the professional interviews, and CAB conducted community interviews. The interviewers shared the vignettes and associated questions on screen and read them aloud at the request of the interviewee. All interviewees were offered $100 in compensation for their time.

### 2.5 Data analysis

We used deductive and inductive elements for qualitative data analysis. First, we transcribed interview audio recordings, and study team members reviewed draft transcripts for accuracy and to remove identifying information. Deductive qualitative data analysis began with the iterative development of a thematic codebook based on concepts from the Public Health Ethics Framework and the topical content of the vignette twists. Two team members (ST and MW) independently coded each interview transcript using Dedoose. They met regularly to refine the codebook, compare coding, and explore differences.

After deductive theme development and consensus on coding was achieved, the team used Google NotebookLM, a generative artificial intelligence program restricted to analyzing user-provided sources, to create synthetic summaries for collections of interview excerpts that had been thematically coded by the study team. JWK independently created synthetic summaries for two themes to compare with and validate the NotebookLM output. Additionally, JWK and CAB checked the accuracy of these summaries, including quotations, via embedded hyperlink references to the coded interview transcripts used to generate the summaries. The authors identified and reconciled discrepancies, omissions, and interpretive errors in the generative summaries using the source documents. Through this inductive process of data validation, the authors identified emergent themes that spanned the summary documents. We present results organized around these emergent themes. We indicate the relative frequency with which specific themes, ideas, and beliefs were expressed by interviewees using qualifiers like “several” and “many” because of our small sample size and concern that explicit enumeration may be misleading as our semi-structured interviews diverged in content due to interviewee knowledge and interests. This approach aligns with recommendations for how to report qualitative data (18). The study team retained the NotebookLM prompts and synthetic summaries in the study database along with interview transcripts.

## 3 Results

### 3.1 Interviewees and WBDS familiarity

We interviewed 13 professionals from February through April of 2023 (interview dates appear in Table 1). We interviewed 8 community members from July to November of 2023 (interview dates appear in Table 2). The professional interviewees worked in the field of wastewater, implemented WBDS programs, or contributed to the evaluation of the ethical, legal, and social implications of WBDS. Table 1 provides additional information about the professional interviewees. Community interviewees encompassed a range of professions, and none had occupational experience with wastewater, public health, or ethics. A few community interviewees reported awareness of WBDS, but none had in depth understanding of the practice. Table 2 has additional information about the community interviewees.

**Table 1:** Characteristics and timing of the 13 professional interviewees.

Interviewee number	Interview date	Field	Employment type	Experience with WBDS	Gender
1	February 24, 2023	Microbiology	Wastewater utility	Implementation	Male
2	March 9, 2023	Engineering	Nonprofit technical organization	Technical guidance	Female
3	March 9, 2023	Engineering	Wastewater utility	Implementation	Male
4	March 10, 2023	Statistics	Health Department	Implementation, Scholarly study	Female
5	March 27, 2023	Environmental health	Academic	Scholarly study	Female
6	March 28, 2023	Public health ethics	Academic	Scholarly study	Female
7	March 28, 2023	Environmental health	Academic	Scholarly study	Female
8	April 5, 2023	Ethics	Academic	Scholarly study	Male
9	April 6, 2023	Public administration	City government	Implementation	Female
10	April 10, 2023	Epidemiology	Academic	Implementation, Scholarly study, Technical guidance	Male
11	April 11, 2023	Law	Academic	Scholarly study	Female
12	April 19, 2023	Engineering	Academic	Implementation, Scholarly study	Male
13	April 21, 2023	Law	Academic	Scholarly study	Female

**Table 2:** Characteristics and timing of the 8 community interviewees.

Interviewee number	Interview date	Familiarity with WBDS	Political identification	Educational attainment	Living environment	Gender	Age band	Home state	Employment status
1	July 24, 2023	Moderately Familiar	Moderate	Finished College	Suburban	Male	59–77	Delaware	Retired
2	July 25, 2023	Minimally Familiar	Moderate	Some College	Suburban	Male	44–58	Washington	Employed Full-time
3	August 14, 2023	Minimally Familiar	Moderate	High school	Rural	Male	29–43	Louisiana	Employed Full-time
4	August 14, 2023	Minimally Familiar	Liberal	Some College	Suburban	Female	44–58	Texas	Retired
5	August 14, 2023	Minimally familiar	Liberal	Finished College	Suburban	Male	59–77	Colorada	Retired
6	August 18, 2023	Minimally familiar	Liberal	Finished College	Urban	Female	59–77	Florida	Employed Part-time
7	October 10, 2023	Minimally familiar	Liberal	Some College	Suburban	Female	29–43	North Carolina	Employed Full-time
8	November 15, 2023	Moderately Familiar	Moderate	Finished College	Rural	Male	59–77	Wisconsin	Retired

### 3.2 Overall receptivity to wastewater surveillance

Community interviewees expressed few apprehensions about wastewater surveillance, and many supported the idea, noting that it could offer quality data for areas tested. Professional interviewees were also overwhelmingly supportive of wastewater surveillance. They noted the need for an established ethical framework for determining when and how to utilize wastewater surveillance, and the need for clear protocols and/or decision trees for public health agencies using this type of surveillance. Wastewater surveillance experts were more able to identify specific risks of testing and potential concerns, but all endorsed it as a useful public health tool. Table 3 compares professional and community interviewee perspectives on WBDS across thematic areas.

**Table 3:** Summary and concordance of professional and community interviewee perspectives on ethics of wastewater-based disease surveillance organized by themes.

Theme	Professional perspectives	Community perspectives
Overall receptivity	Overwhelmingly perceived WBDS to be a highly valuable, life-saving public health tool	Strong support for “Brilliant” and “really intelligent” idea of WBDS
Transparency and communication	Public information-sharing and community buy-in needed prior to implementation	“Let people know what’s going on” – share surveillance aims and results with community
Privacy, sample size, and identifiability	Concerns regarding small-scale surveillance due to privacy risks, potential community stigma, and lower data reliability	Interest in localized, granular surveillance to aid individual decision-making while maintaining privacy
Human DNA analysis	Some concern of future re-analysis of banked specimens for human DNA	Some skepticism regarding organizational assurances related to (not) testing human DNA in wastewater
Equity and fairness	WBDS data less biased than traditional surveillance data. Risk excluding unsewered populations. Potential for unfair actions if communities identified negatively.	Inclusive – “everybody’s waste is going to the same system.” Risk of missing unsewered populations.
Benefits of WBDS	Benefits include individual privacy protection, data independent from healthcare-seeking behaviors, cost-effectiveness, rapid results, and early warning capabilities	Benefits include early warning capabilities and inclusive data independent from healthcare-seeking behaviors.
Limitations of WBDS	Inherent and technical limitations include viral shedding variability, detection challenges at low levels, and the risk of data misinterpretation.	No technical limitations noted but stressed importance of local context for data interpretation
Clear purpose and actionable outcomes	Transparent, legitimate, and actionable purpose with community benefit necessary - “doing it for the right reasons.” Explicit public health duty to act on data, particularly to protect vulnerable populations	Use WBDS technology “intelligently and professionally.”
Data ownership	WBDS is a public good, data belongs to the community with public health agencies as primary stewards. Data-use agreements needed when using private labs to mitigate profit motives.	Some concerns about the commercial exploitation and future misuse of banked specimens.
Law enforcement	Universal concerns with sharing illicit drug wastewater data with law enforcement	Concerns that law enforcement would use WBDS data for punitive action
Illicit drug surveillance	Substantial concerns with WBDS for illicit drugs at high schools. Risks of loss of public trust and punitive actions heavily outweigh public health benefit.	Broad support of illicit drug WBDS at high schools with caveats that data used to raise awareness and provide school-level resources
Stigma	Some concerns for WBDS for highly stigmatized conditions, such as sexually transmitted infections. Consider whether to share data publicly for vulnerable populations (e.g., incarcerated)	Few concerns regarding community stigmatization. Endorsed sharing wastewater data publicly under all circumstances.
Oversight; legal and ethical frameworks	Broadly endorsed institutional adherence to established guidelines, like the WHO surveillance framework, and use of structured ethical tools, such as “decision trees”	Need for oversight framed pragmatically in terms of “making sure” or “guaranteeing” specific protective outcomes rather than identifying or proposing specific structures.

WBDS, wastewater-based disease surveillance. The row color scheme indicates the qualitative concordance between the perspectives of the two interviewee groups. Green indicates high concordance, yellow indicates mixed concordance, and red indicates discordance. Heavy borders for a box indicate that the perspective may be influenced by limited technical knowledge of WBDS.

Many of our community interviewees contrasted wastewater testing with other forms of disease testing they had experienced; for example, COVID-19 nasal swab tests came up in several community interviews. When we conducted our interviews, many members of the public had recently encountered public health actions in a way unlike anything they had experienced, such as contact tracing, quarantining, and the closure of public spaces. Our community members compared wastewater surveillance favorably to the public health measures they had recently experienced. One professional interviewee (5) anticipated this receptivity, noting that less political will would be needed to maintain WBDS because “*testing wastewater … is not annoying to people*,” whereas COVID testing of individuals is “*politically very unpopular and expensive*.”

### 3.3 Conditions that make wastewater surveillance okay

Interviewees from both groups identified multiple values and commitments they thought should underlie any use of wastewater surveillance. We asked interviewees in a colloquial manner to describe what they thought was necessary to make wastewater surveillance “okay.” These requirements included transparency, effective communication, data privacy, legitimate purpose, responsible data use, public oversight, equity, and community engagement. Several professional interviewees identified the importance of following existing public health ethical frameworks, such as the WHO surveillance framework, along with pertinent US law related to data disclosure. Many community interviewees also identified the importance of oversight, although they did not specify a responsible party. Both professional and community interviewees articulated the need for transparency with communities regarding wastewater surveillance programs – their purpose, what is being tested, how the data will be used, and how it will not be used. Several professional interviewees noted the need to communicate this information through various channels and using language understandable to the intended audience.

Using a public process in the development of a wastewater surveillance program, including appropriate professional expertise and community input was mentioned by a couple of professional interviewees. Maintaining privacy and data security were also highlighted by interviewees as necessary components of an ethical approach to wastewater surveillance to protect individuals and communities from loss of privacy and stigma, with specific concerns about not sharing potentially sensitive health information, like illicit drug levels in wastewater, with law enforcement.

### 3.4 Transparency and communication

Transparency with the public was mentioned frequently by community and professional interviewees alike. One professional interviewer explained: “*I think public health should be extremely careful and very transparent about what it is they are going to test and make sure that when they bring that to the community they are able to be clear about what the benefits are, what the risks are, and what public health action would come as a result of it.”*(Professional 6). Many community interviewees voiced similar thoughts, emphasizing that what is most important is to “*let people know what’s going on*.” (Community 16).

In general, community members expressed positive opinions of wastewater testing with minimal prompting. However, this approval was often verbalized in conjunction with a requirement for honest communication with the public and transparency about the aims of wastewater testing. Community members envisioned wastewater testing accompanied by accurate and complete information, both in terms of clarity about what testing was occurring as well as full sharing of wastewater testing results with communities tested. Community members associated commitments to transparency with a concurrent need for guarantees that data would not be used punitively against the community being tested. One community member explained: “*they should make people aware of what’s going on. And that no one’s going to get in trouble for fentanyl use or the pregnancy hormones in their urine or anything else. It’s a little frightening because [of what] somebody in charge with ill intent can do. I can see some people being super paranoid about this*.” (community 16) This type of speculation that ‘someone else’ could be concerned about wastewater testing, but they themselves were not, happened several times in our community interviews.

Professionals similarly endorsed wastewater testing as effective for various public health aims, but they stipulated, just as community members had, the need for information-sharing with the public. Many professionals emphasized the need for community buy-in prior to beginning wastewater surveillance. In general, when asked what conditions needed to be in place prior to wastewater testing, professionals, like community members, identified transparency as a necessary condition. Many professionals spoke in detail about mechanisms to achieve effective community communication, identifying strategies such as using redundant forms of communication, using simple, non-medical language in communication, and being consistent and honest when sharing plans about wastewater testing.

### 3.5 Privacy, sample size, and identifiability

The interview vignette that probed for an appropriate population scale for WBDS revealed a range of opinions. Several community interviewees expressed significant interest in localized (neighborhood and even apartment building level) surveillance, noting the value that localized information on disease trends would provide for individual decision-making. Community interviewee 16 stated “*The more specific they can get, the better. Like if somebody called me and said, “We’ve tested your apartment building and somebody there has COVID,” I would appreciate the information.”* Another community interviewee (19) noted “*And I do not know about. concerns for privacy? I do not know. How that would even be applicable because, I mean, after your waste goes into the sewer, there is no privacy. It’s like, you know, putting your trash on the street*.”

Multiple professional interviewees described the benefits of more localized testing to support geographically focused public health interventions. Examples included locating testing or vaccination sites in communities with many individuals at higher risk of severe illness, like those living in nursing homes. A couple of professional interviewees shared anecdotes about their participation in localized wastewater testing projects. They believed these projects increased vaccine uptake in those communities through targeted messaging campaigns in response to surges in virus burden detected in wastewater. However, professional interviewees also expressed concerns with smaller scale testing, notably perceived risks to privacy, the resources needed, potential for community stigma, reliability of surveillance data at finer scales, and the public health justification for small resolution wastewater surveillance. Interviewees questioned the advantages of smaller scale testing (e.g., testing representing ~500 individuals) and whether the data from such granular surveillance would be more useful than from larger scale (e.g., >3,000 individuals). Several interviewees mentioned that the useful geographic threshold may vary during a disease outbreak with smaller scale WBDS potentially useful for a highly dangerous virus. One interviewee highlighted the importance of co-developing wastewater surveillance plans with communities to meet their needs, which could vary from surveilling a handful of individuals on the International Space Station to millions of individuals in an urban sewershed.

When asked about human DNA in wastewater, several professional and community interviewees expressed concerns about the potential of identifying individuals. While the technical feasibility of this was questioned by some professionals, it was acknowledged that it would likely be possible soon and could be used to re-analyze banked wastewater specimens in the future. A couple of community interviewees expressed skepticism regarding assurances from organizations conducting WBDS that they would not test for human DNA and had concerns that this could result in tracking individuals without their consent.

### 3.6 Equity and fairness

Many professional interviewees reflected that “fair” has many potential meanings when prompted to consider fairness and equitability in wastewater testing. Professional interviewees tended to view fairness from the lens of unbiased and valid data collected with the intent of informing public health interventions, while community interviewees viewed fairness from the perspective of privacy and community awareness of WBDS. Additionally, one professional was concerned about equitable national investment of resources for WBDS to provide nationally representative data and local information to communities. However, this same interviewee acknowledged that WBDS may provide more equitable surveillance information than traditional case-based surveillance which may omit individuals with limited access to healthcare.

Professional interviewees recognized WBDS as providing a relatively fair (unbiased) assessment of disease levels in a community. As one professional interviewee (13) noted “*although there are equity problems with wastewater surveillance, there are huge equity problems with other means of surveillance of all kinds. I think it does provide information about folks who do not show up in the clinical care system … and that, you know, that’s really valuable.*” A community interviewee (20) echoed this view stating, “*you are going to get everybody’s wastewater not depending on, you know, people reporting and maybe people at one situation are more likely to report than another.*” However, there were many professional interviewees who expressed concerns about the exclusion of unsewered populations from WBDS, such as unsheltered people and those living in rural areas. “*There could be groups treated unfairly, like if you are testing in one area and not another area of your community or you are making decisions that are disparate amongst a community because of what a singular wastewater data set is telling you.*” (Professional 9). Interviewees generally felt that WBDS was a fair (representative and valid) way to measure disease levels but that unfair actions could result if certain groups or communities were singled out negatively because of wastewater results.

Community interviewees also felt WBDS was a fair way to measure disease in a community. “*Where I live, everybody’s waste is going to the same public sewer system, and they would all be included.*” (Community 20) There was also recognition that certain populations might not be well represented by or benefit from WBDS. “[Wastewater testing] *would be limited to communities that actually have a wastewater treatment system, which would tend to probably over represent people around cities, suburbs, that type of thing. Whereas in more rural areas there’s, in many cases there’s no public sewers, or limited public sewers. Most of it’s on septic tanks and everything. So, I think … certain areas would be underrepresented.*” (Community 14).

### 3.7 Benefits and limitations of WBDS

Professional interviewees believed WBDS for COVID-19 to have high value, describing it as a “*fantastic tool*” (Professional 3) and as “*really important and very valuable and will save lives*.” (Professional 4). One interviewee (Professional 8) noted that “*public health authorities and their partners … have a moral obligation to conduct this kind of surveillance in their community*.” Community interviewees also expressed positive reactions to WBDS as a public health surveillance tool, using expressions like “*good idea*,” “*really intelligent*,” and “*brilliant*.”

All professional interviewees identified advantages and benefits of WBDS, particularly related to COVID-19 surveillance and in contrast to clinical testing. These advantages include protecting the privacy of individuals, data that is independent from healthcare seeking and testing behaviors, cost effectiveness for large scale surveillance, ability to detect asymptomatic cases, and efficiency (speed and low cost). Some interviewees mentioned that WBDS provides the only accurate assessment of COVID-19 with the lack of testing and reporting of test results. The ability of WBDS to provide an early warning of disease circulation was another noted benefit, and several interviewees shared anecdotes of how disease signals from WBDS informed preparedness and prevention strategies in schools, hospitals, and specific communities.

A professional interviewee noted that WBDS could play an important role in identifying emerging diseases, such that WBDS data could be paired with syndromic surveillance data from healthcare systems and that sentinel WBDS sites could be developed to identify diseases entering the US from other locations. However, several professional interviewees noted the importance of using scientific criteria to select appropriate pathogens for WBDS and the challenges with interpreting wastewater data for certain diseases like polio.

Community interviewees had fewer insights into the potential benefits of WBDS. Several noted it has value as an early warning system for communicable diseases and provides information on everybody, not only people choosing to get tested. Another interviewee commented that WBDS data could inform efficient resource allocation.

Most professionals also noted inherent limitations to WBDS and suggested it should augment but not replace traditional disease surveillance data. Limitations mentioned included not knowing who contributed to the wastewater sample, detection of viruses at low levels and at smaller scales, and individual variability in COVID-19 viral shedding. A professional interviewee noted concerns about the potential to misinterpret or misunderstand WBDS data – by individuals and health officials. Interviewees mentioned the importance of interpreting wastewater data within the context of these limitations, the local context (e.g., potential effects of tourism), and the use of statistical techniques to minimize data limitations. Community interviewees did not specifically comment on the technical limitations of WBDS; however, they mentioned the importance of including local context and interpretation of the wastewater data when communicating with the public.

### 3.8 Clear purpose and actionable outcomes

Many professional interviewees noted the importance of having transparent and legitimate reasons for conducting wastewater surveillance – “*doing it for the right reasons*” (Professional 3). This was echoed by community interviewee 20 who stated, “*If it’s used intelligently and professionally, I think it’s great*.” Various wastewater surveillance scenarios discussed with professional interviewees yielded responses that highlighted the importance of defining the purpose and actionability of the surveillance activities. “*Any type of public health surveillance is meant to provide understanding. And so, once that understanding is had.the decision there is then what to do with the data or how to act, and that’s always a political choice. Now how will the local health departments use that data? I hope in the future it gets used more to help to do more tailored interventions. More precise interventions*.” (Professional 10) This theme also emerged during discussions of testing wastewater for human DNA; both community and professional interviewees mentioned that there would need to be a clear public health reason for testing DNA in wastewater.

Several professional interviewees noted that public health agencies had a duty to act on wastewater information, specifically in scenarios where vulnerable populations, like those living at long term care facilities, were under surveillance. Professional interviewee 3 said “*So in terms of the responsibilities, I think it’s having clear goals of what you are trying to accomplish, or what questions you are trying to answer when you get to these specific levels or specific facilities, or that you are doing it for the right reason.*” Professional interviewees also noted that there should be criteria used to decide which additional health issues should be measured in wastewater and suggested that one criterion should be the actionability of the data. One Professional interviewee (1) took it a step further and stated “*I definitely am now saying, well I do not want to do anything if there’s going to be no action on it. I want to do it if this is actually going to have a benefit to my community.*”

### 3.9 Data ownership

Many professional and some community interviewees had concerns about corporate testing of wastewater as well as access to its results. Several professional interviewees identified public health surveillance as a public good with health departments and ultimately communities as the preferred data stewards. A professional interviewee (9) summarized this view by saying “*Regardless of which lab is doing the testing, that data, I think, belongs to the community that’s providing it, providing the wastewater. Believe it or not, wastewater is a community asset, and I go back to it’s the people’s data.”* Interviewees expressed concerns about profit motives driving decision-making if WBDS were to be conducted by commercial entities. Some professional interviewees acknowledged the need for contracting with private labs for wastewater analysis and emphasized the importance of establishing clear data use agreements that provide clear guardrails for the type of testing, data use, and sample storage. Community interviewees had fewer opinions regarding the type of entity testing wastewater if there was oversight. A few community interviewees had concerns about the misuse of wastewater data for commercial benefit, and one individual had concerns about the potential for future misuse of banked specimens given anticipated technological advances, specifically related to DNA analysis.

### 3.10 Law enforcement

Interviewees were asked what they thought of a scenario in which a high school decides to test their wastewater for illicit drugs. Most community interviewees indicated strong support for this measure, with certain caveats. One interviewee with a child currently in high school remarked, “*I’m for it. Let us do it. I would love to have that*.”(Community 17) Several interviewees without children anticipated this sort of parental approval, with one stating, “*I think most parents would love it*.”(Community 14) These expressions of support were generally coupled with a stated desire to keep children safe and a recognition that drug use in high school was a problem in need of a solution. For most community members, however, this support was conditional; many interviewees added caveats or concerns to their claims of support without prompting. Concerns raised include the invasion of privacy this poses for students, the potential liability this poses for schools in possession of this information, and the possibility of law enforcement using this for punitive rather than reformative purposes. Many community members stipulated that their support for testing high schools for illicit drugs was limited to use-cases described in terms of sharing information, increasing awareness, and providing resources. At least one community interviewee (14) anticipated legal complexities, explaining: *“I would certainly, certainly support it. … The question then is what responsibility would the school have to program around it [and] communicate with parents? You know, prevention type programs and everything else? How does that raise, you know, the accountability of the individual organization and what type of liability might it create for those organizations?”*

In general, professional interviewees were much more apprehensive about testing high schools for illicit drugs than community members. No professional interviewee endorsed this use-case for wastewater testing. Many of them believed that the risks outweighed any potential benefits. They cited concerns related to public trust, feasibility, and potential misuse of information as reasons to be cautious about testing high school wastewater in this way. One explained: *“I think it would take more information to persuade me that the benefit risk cost calculus came out positive there. On the risk side it’s hard to imagine that many schools could help themselves in the sense of not wanting to go find the people who were doing the drugs and discipline them if they had that information and they were seeing the things were going up. On the benefit side, I just think that there are other ways of figuring out levels of drug use in the school, none accurate in their entirety, but available, and that all schools ought to be getting interventions from the Health Department around prevention of drug use.”* (Professional 13).

This same professional interviewee anticipated several difficulties related to feasibility and efficacy. While they were unsure of whether schools would be legally required to get permission prior to testing, they explain that: *“My expectation would be that the school would inform parents. At least in my community, they inform the parents about everything. But the problem here is that telling in and of itself could undermine the testing, because it’s very easy for kids to just not poop at school. It’s one of the first things I learned on [a] wastewater committee: kids do not poop at school. So that’s just another reason why this does not particularly make sense to me as a public health intervention*.” Other professional interviewees emphasized the need for transparent, open communication with all stakeholders and clearly defined objectives prior to considering this. Overall, they were unable to imagine a path to endorsing this as a wise public health intervention. One interviewee explained that even if quality information was gained, the potential damage to the public was too great. “*And so, I do not mind missing a little bit of data in exchange for a steady process by which we build it sustained trust. Otherwise, it will not matter that we are right. It’s a very fragile relationship with some populations that are traditionally either discriminated against marginalized or stigmatized.”* (Professional 8).

Concerns raised by both professional and community members in response to this hypothetical scenario reveal a consistent apprehension related to wastewater testing data being used by law enforcement to implement punitive measures in the community. Follow-up questions related to how schools might appropriately utilize information about the presence of drugs in wastewater revealed strongly negative views of individualized applications, such as urine drug screens or mandatory backpack checks. Community and professional interviewees preferred system-level approaches such as targeted education about drug misuse, and addiction support resources. Most community members voiced that urine drug screening would be a misuse of wastewater data, expressing concern that it would be “*a little too far*” and that it may deter students who use drugs from going to school at all. Professional interviewees also emphatically opposed mandatory urine drug screens in response to wastewater testing results, with one fearing it was unconstitutional.

Overall, community members and professionals alike supported non-individualized responses to wastewater drug testing, such as education about addiction and voluntary substance abuse counseling. Some voiced concerns about the effectiveness of these measures, though, and others suggested these should be provided to all students regardless of wastewater testing results.

### 3.11 Stigma

Few interviewees reported specific concerns related to wastewater testing and community stigma. Concerns that were reported included the potential to weaponize wastewater data against certain races, and the potential to learn about criminal activity in a location by testing for illicit drugs. Many community members were not concerned about the potential for stigmatization from wastewater testing for different diseases, suggesting that if health departments were transparent about what was being tested and the results of those tests, there was little issue. When asked about vulnerable populations, community members were unable to think of specific risks from wastewater testing, although in other contexts, they expressed concerns associated with illicit drug testing and law enforcement access to the data. Professional interviewees were more likely to note stigmatization risks of wastewater testing, such as associations of certain diseases with specific groups due to high disease prevalence in wastewater, especially already stigmatized diseases such as sexually transmitted infections. Perhaps because of this, one professional interviewee stated explicitly that wastewater testing done on vulnerable populations should not be made public, whereas all community members endorsed making data public under all circumstances.

### 3.12 Oversight and legal and ethical frameworks

Interviewees occasionally used moral language to speak about the role of wastewater testing in society; for example, one professional (8) stated that “*public health authorities and their partners in academia and government have a moral obligation to conduct [WBDS] in their communities*.” Most often, though, both professional and community interviewees explored ethics as it related to a need for oversight, legal constraint, and professional ethical frameworks to guide wastewater testing.

For most community members, the need for oversight and regulation was communicated in terms of “making sure” that certain things did not happen or “guaranteeing” that certain requirements were in place, rather than explicitly identifying the source of these constraints. Community members expected oversight to ensure transparency and communication with those being tested, proper data ownership and privacy, and protection from wastewater data use by law enforcement or for punitive measures. When speaking about community input, community interviewees stated the importance of letting people know what is going on, sharing wastewater testing results, and telling the public when it is happening.

Professionals often mentioned the need to review existing guidance to ensure wastewater testing complied. One interviewee mentioned the WHO guidelines on public health surveillance and indicated that the US has existing laws related to the same. Several professionals suggested the need for a “decision tree” or tool for public health agencies to use when planning wastewater surveillance that would guide them both ethically and legally. Another professional interviewee (8) suggested that a “*Public Health Ethics Review Committee*” be established to ensure that vulnerable populations were protected and to weigh the benefits versus possible harms of wastewater testing in various scenarios. Another professional interviewee (2) focused on the need for clarifying who should provide this guidance, saying, “*What questions [do] the public health department need to answer?. How do you engage community groups?. I do not think it’s the utilities job to do everything, answer all the questions. I do not think the Health Department should answer them all on their own. We need some kind of framework and decision tree about how to decide to put a program in place and expand it*.” Another spoke in detail about what she believed an ethical framework ought to include:

“*So, I mean my fundamental piece on this is just that that having a system to do wastewater testing or wastewater surveillance I think, is good. I think public health should be extremely careful and very transparent about what it is they're going to test and make sure that when they bring that to the community, they are able to be clear about what the benefits are, what the risks are, and what public health action would come as a result of it. So, I think it can't be so… So that's one thing. The other thing is it needs to be in the hands of a public health entity that that system and the data this the system collects, and then you know what happens with holding those data, analyzing those data, using those data. That needs to be in the hands of public health, not a private entity.”* (Professional 6)

In general, both professionals and community interviewees identified the need for safeguards to ensure things like transparency with the public, proper data ownership, and protection from data misuse. Both groups indicated support for oversight and community input prior to, during, and after wastewater testing. Professional interviewees were more likely to have specific suggestions about the ethical frameworks required as well as the appropriate source of legal or ethical oversight. However, there was strong consensus between both groups of interviewees about the aims of this oversight and the necessity of it.

## 4 Discussion

This empirical study adds professional and lay community member perspectives regarding the ethics of WBDS to the growing body of literature in this field. Community voices have been absent from published ethical explorations and guideline developments. Our study revealed widespread support for wastewater testing. Most community interviewees were highly receptive to wastewater surveillance despite minimal prior familiarity with it. Our study suggests that communities are likely to find wastewater surveillance acceptable if certain ethics practices are put in place.

The broad support for WBDS voiced by community interviewees was also found in national and regional surveys of community members’ knowledge, attitudes and beliefs regarding WBDS. A 2023 nationwide survey found that 91% of respondents supported or strongly supported WBDS for infectious disease surveillance (14); a study in Appalachian adults found that 72% of respondents supported WBDS. Several characteristics of WBDS may contribute to its acceptance by the public, including it being anonymous and less intrusive, which differentiate it from clinical testing.

Interviewees expressed interest in the potential benefits of localized wastewater surveillance. The geographic scale of WBDS was frequently identified as a potential concern due to potential loss of privacy and community stigma (19, 20). The CDC’s National Wastewater Surveillance System suppresses data from locations with fewer than 3,000 individuals for this reason. However, other jurisdictions, such as the city of Houston, publicly report school-level data weekly (21). In our sample, community members believed that localized wastewater testing could obviative the need for more invasive measures like lockdowns and widespread quarantining. They identified the information that wastewater testing provides as empowering their ability to assess risk and take preventative measures. Professional interviewees endorsed the potential benefits of localized testing as well, if measures to ensure individual anonymity were prioritized.

Our responses suggested little politicization of wastewater testing, as far as no community members attached larger beliefs about the government or even COVID-19 to their views of wastewater testing. We interpreted several community members to be anticipating such politicization when they commented that other individuals may have concerns about wastewater testing or that they could imagine someone objecting to it, although our interviews did not reveal interviewees’ intent. It is possible, for example, that they themselves had similar apprehensions but felt uncomfortable sharing them, or that they felt obliged to identify concerns of some sort given the nature of our questions. It is also possible that our sample of community members, all of whom had agreed to be interviewed about the topic of wastewater, self-selected for positive perceptions of the topic.

We found that interviewees had mixed opinions about using WBDS to monitor illicit drugs, which may have stemmed in part from the high school setting of the vignette. Community interviewees generally supported WBDS for illicit drugs, and several interviewees shared how their lives had been touched by the opioid pandemic. With one in three US adults reporting knowing someone who died of a drug overdose in the US, more than 60% of Americans view addiction as an extremely or very important policy priority (22). Thus, many people may be receptive to new ideas on how to better understand and identify illicit drug use, such as through wastewater surveillance. Within the context of the interview vignette, community interviewees felt the potential benefits would outweigh the potential burdens when there were not individual level impositions that resulted from WBDS, such as backpack searches or urine drug tests.

Professional interviewees had more reservations about WBDS for illicit drug surveillance. Some concerns were likely related to the vignette focusing on school surveillance, which introduced questions about school stigma and parental consent that may not exist for municipal-level drug surveillance. Professional interviewees’ main concern was law enforcement accessing the wastewater surveillance drug data and using it in punitive ways. Several professionals strongly articulated that illicit drug use is a health issue and not a criminal problem and hence wastewater surveillance should be used to support public health responses. These concerns map to several elements of the Public Health Ethics Framework, namely the goals of the proposed program, the potential burdens, and fair implementation. One professional shared how her jurisdiction used a community town hall meeting to communicate plans for illicit drug wastewater surveillance to facilitate transparency and maintain community trust. Law enforcement was not included as part of the multidisciplinary team, although the interviewee acknowledged that they would be able to view community level data on the public facing dashboard.

One goal of our interview study was to consider how professionals’ and community members’ views might fit into the six steps of the Kass public health ethics framework, steps intended to help public health practitioners identify and analyze public health initiatives through an ethics lens. The first step of this framework examines the public health goal of the proposed initiative. Many interviewees from both groups were clear that wastewater surveillance should provide actionable information. This is consistent with the framework’s requirement that public health interventions ought to be tied explicitly to a plan to reduce community morbidity or mortality (i.e., not merely to gain information or research population health). Of note, this study did not examine the ethical considerations of wastewater analysis for research purposes. Research using wastewater samples raises additional ethical considerations that have been explored by other scientists (13, 19, 23, 24).

The second step of the framework requires that the activity being proposed has evidence that links it to achieving (at least in part) the stated goal. Professional interviewees all endorsed the effectiveness of wastewater testing for achieving public health goals. Both community and professional interviewees endorsed that wastewater surveillance is highly effective for monitoring disease presence and trends. Several professional interviewees shared how wastewater surveillance had supported localized public health interventions, like immunization campaigns, which would benefit public health. Several community members reflected on the impact that wastewater surveillance data would have on their own choices, and the potential for them to make more informed decisions to protect their own health.

The third step of Kass’s public health ethics framework assesses known or potential risks and burdens of an intervention. Community members perceived few burdens from wastewater surveillance and understood these to be comparatively less than other disease testing and public health surveillance activities they had experienced. Burdens that community members identified generally focused on (1) the prima facie invasion of privacy that wastewater testing represents, which was not of high concern, particularly in relation to alternatives; (2) the potential for misuse by groups not transparent about their use of data, which was of high concern, and (3) the potential for law enforcement to use wastewater data to justify more invasive forms of surveillance, which was of high concern. Professional interviewees shared concerns about the potential for law enforcement misuse and were sensitive to the need for transparency to avoid public mistrust.

Interviews provided useful information about how to reduce burdens for communities, which is step 4 of Kass’s ethics framework. Community members were less likely to view wastewater surveillance as overly burdensome when accompanied by transparency and clarity about who is performing the surveillance, and when strong protections were in place to ensure that the data produced is only accessed by public health entities who answer to the public. Most community members suggested that transparency about the purpose of public health surveillance would similarly reduce the perceived burden placed upon their communities. In general, they relayed few concerns about burdensomeness, instead noting wastewater surveillance’s relative lack of imposition and the fact that they themselves would be uninvolved in the process of data gathering.

The fifth step of the framework assesses the fairness of intervention implementation. Most interviewees, professional and community, endorsed wastewater testing as fair or more fair than other forms of public health surveillance. However, at least one professional noted concern for individuals not included in wastewater surveillance, for example those on septic tanks or otherwise with limited interaction with public sewer systems. This interviewee noted the potential for groups who do not contribute to wastewater data to still experience the burdens of it, both via their tax dollars spent on these programs and potential actions taken due to wastewater data. This person stated that these fairness concerns did not outweigh the benefits wastewater surveillance offers. Community interviewees did not share fairness related concerns with wastewater surveillance implementation even when specifically asked about such concerns.

Our interviews yielded a consensus that the burdens of wastewater testing could be offset by community benefits. The sixth step of the framework examines how the benefits and burdens of a program can be fairly balanced. Mitigating community burden, according to interviewees, is best done by implementing wastewater surveillance with transparency, ensuring it is done by government agencies answerable to the public with strong protections against private sector misuse and has identifiable public health goals associated with the program.

Both groups of interviewees endorsed the importance of transparency and communication with communities regarding the occurrence, aims, and findings of WBDS. This element is not explicitly described in the Public Health Ethics Framework, albeit the sixth step embraces societal discourse to balance benefits and burdens. A recently published scoping review of the ethics of WBDS found that effective communication was the third most frequently discussed ethical theme (10). A recently published Association of State and Territorial Health Officials report on ethical considerations of WBDS identifies community engagement as critical and explains engagement as contributing to the ethical goals of “*acknowledging past harms, reducing the potential for current and future exploitation, and helping to ensure that policy, program, and research goals respond to the needs, priorities, and circumstances of those most affected by public health problems*.”(page 8) (9) Further, the report describes the importance of effective public communication to mitigate privacy concerns and prevent stigma by describing the goals and benefits of WBDS and planned uses of the data. These communication elements were reinforced by our community interviewees, including a desire for assurances from the agency conducting WBDS that the data would not be used punitively.

Communication undergirds community transparency and enables the coordination of the multiple entities likely to be involved in the implementation of a WBDS program for public health. A growing body of literature explores communication approaches specific to the field of WBDS. For example, a National Academies of Science, Engineering, and Medicine report on WBDS (25) provides a framework for collaboration between public health agencies, wastewater utilities, analytic laboratories, and the scientific community (Figures 4–2, page 115); however, public communities are missing from this framework. The WBDS ethics framework proposed by Bowes et al. (13) recognizes the importance of transparency and community communication within the domains of “awareness,” “public decision-making,” “external data sharing,” “community values,” and “right of inspection” through a process of “continued community outreach and engagement.” The City of Tempe exemplifies one approach to community transparency and communication with its Wastewater Biointel Program which monitors COVID-19 and opioid levels in city wastewater (26, 27). Through townhalls, news stories, and public facing dashboards Tempe officials sought community input and shared findings. The NASEM report provides additional examples of WBDS communication strategies used by public health agencies with communities (25).

Our study has notable strengths and limitations. Our sample of community and professional interviewees was small, and we may not have obtained saturation in all thematic areas, particularly regarding community perspectives. However, we solicited a broad range of community interviewees through a national survey and purposefully selected a sample of respondents based on their sociodemographic characteristics to obtain a diversity of opinions. Notably, we achieved a diverse community sample in several areas; five interviewees identified as male and three as female, each was in a different state, and they spanned a range of ages. Community interviewees represented suburban, urban, and rural living environments, as well as varying levels of education attainment. We aimed to recruit individuals with a diverse range of political views. However, our purposeful sampling based on self-described political ideology yielded only “liberal” and “moderate” participants. The reason for the poor response rate from individuals identifying as “conservative” was unclear; however, an increasing amount of time had passed between the initial Qualtrics survey and our purposeful emails to conservative-identifying community members – a span of several months, as opposed to several weeks for our initial batch of email interview invitations. In summary, email invitations sent relatively soon after the Qualtrics survey had relatively high response rate, whereas later outreach efforts to diversify our interview sample were mostly futile.

Professional interviewees were identified through their contributions to WBDS reports and papers, which likely introduced a selection bias toward individuals supportive of WBDS. However, the authors made explicit efforts to also interview professionals with limited previous WBDS exposure (e.g., bioethicists) and individuals who had published critiques of WBDS. The use of hypothetical vignettes in a semi-structured interview guide provided context and a framework to explore potential ethical concerns related to WBDS but also may have constrained interview responses within the boundaries of the vignette details. For example, the vignette describing illicit drug surveillance at a high school may have curtailed interviewee examination of WBDS for illicit drugs in other contexts, such as at the municipal level. The interviews were conducted at the tail of the COVID-19 pandemic, and public and political sentiments toward public health activities may have since shifted, necessitating follow up studies with community members.

The growth of WBDS during the COVID-19 pandemic has led to multiple ethical guideline proposals (9, 11, 12). Our empirical study contributes the voice of lay community members to this process along with professional opinions. Community interviewees overwhelmingly responded positively toward WBDS and expressed few concerns. The ethical values and potential concerns shared across interviewees align with those identified in other recent treatments of the ethics of WBDS, namely privacy, effective communication, governance, clear public health purpose, data ownership/sharing, and fairness. Stigma was of minimal concern to our interviewees in contrast to its frequent appearance in WBDS guidelines papers. Conversely, there was broad concern among interviewees for sharing WBDS data with law enforcement, particularly illicit drug surveillance data that could be used punitively. While continued exploration of public sentiment and concerns related to WBDS and its growing number of use cases is warranted, the support voiced by our community interviewees is encouraging for public health entities considering or engaged in WBDS activities.

## Data Availability

The raw data supporting the conclusions of this article will be made available by the authors, without undue reservation.
