# Supplementary material for: Ethical considerations of wastewater-based disease surveillance—a qualitative interview study
Source: Front Public Health. 2026 Jul 7;14:1860050. doi: 10.3389/fpubh.2026.1860050 (PMC13385672; doi:10.3389/fpubh.2026.1860050)
Supplement: Supplementary file 1 [file Supplementary_file_1.DOCX]

Supplementary Material

**Developing stakeholder-engaged ethical guidance for public health wastewater surveillance**

**Stakeholder Interview Guide**

[*Greet/welcome participant*]

[*Review informed consent, ask for participant questions, and confirm receipt of signed electronic copy*]

Thank you for agreeing to take part in our study about what people think about testing wastewater to learn about the health of a community. We are particularly interested in ethical concerns about this approach. Some people have worried about privacy, fairness, and how wastewater data is used to make public health decisions. Your thoughts on this topic will help our team provide advice for organizations that use this new public health tool. This interview should last about 45-60 minutes.

If there are questions you do not want to answer, that’s fine. Just let me know and we can skip them. I will be voice recording our conversation. That helps me not have to take so many notes as we talk and helps us make sure we hear what you are saying correctly. Do you have any questions for me before we begin?

[*Start recorder*]

1. What is your occupation?
2. How long have you worked in this field?
3. Are you familiar with wastewater surveillance? We know that not everyone has heard of this. [*Probes: What does it mean to you? How did you learn about it?*]

[*Depending on participant’s level of understanding provide this basic description*: Wastewater is in the sewer system. It includes the water from the kitchen, laundry, and bathroom after it goes down the drain. Wastewater has human waste – urine and feces – in it. By testing wastewater samples for germs we can learn about the health of the community connected to the sewer system. Many cities are now testing wastewater to learn about the spread of COVID-19 in their communities. Traditional COVID-19 disease tracking systems count the number of positive clinical tests (nose swabs), patients hospitalized because of COVID-19 infection, and deaths from COVID-19. Wastewater testing can provide information on community COVID-19 levels even when not many people are being tested. Wastewater data may help health departments make decisions on how to keep people safe, for example by identifying hot spots of infection to focus resources like testing and treatment centers.]

Let’s review a made-up wastewater-testing situation. Then I will ask you questions to learn when you think it is appropriate to test wastewater. Please answer from your perspective as a [occupation] and as a member of a community that might be testing wastewater.

[*Share made up scenario*]

Your local TV station runs a story about COVID-19 wastewater surveillance. You learn the local public health department is testing wastewater from your community. Wastewater comes from places like sinks, toilets, and the dishwasher and ends up in the sewer system. The health department is measuring levels of COVID-19 at the wastewater treatment plant twice a week. A public health official says wastewater testing gives us important information on whether COVID-19 is going up or down in the community. They say wastewater testing is important because fewer people are getting tested or reporting home test results. The health department has started testing wastewater at the neighborhood level to target public health interventions. They share the results on a public website. You open the website. Wastewater COVID-19 levels have gone up in the past two weeks in your neighborhood. During this time, reported cases from COVID-19 testing have stayed low.

Can I answer any questions about the made-up scenario?

1. What do you think about testing wastewater to track COVID-19 in a community?
2. What do you see as potential concerns or downsides to doing this? How might your concerns be addressed?
3. What do you see as some of the advantages of using wastewater to track COVID-19 in a community? [*Probe: Some advantages mentioned by others include the early detection of the virus before individual tests are positive and the ability to track COVID variants using wastewater. In addition, wastewater testing uses fewer testing resources to understand what is happening in a community compared to individual testing. Another possible benefit of wastewater testing is getting information on communities that may have less access to or interest in clinical testing. What do you think about these potential advantages of wastewater testing?]*
4. Can you think of ways that the health department could collect similar information about the spread of COVID-19 in the community? Would these approaches be more or less bothersome to individuals or the community?
5. How is wastewater testing a fair way to measure disease in a community? Can you think of people or groups of people who might be treated unfairly because of wastewater testing? [*Probes: How could it be made fairer?*]

Now I’m going to add some twists to the made-up case scenario to see how your opinions might change about testing wastewater.

[Twist 1 - scale] Your local health department proposes testing wastewater at the neighborhood block level – about 500 people - to have a more detailed picture of COVID-19 spread and to better focus interventions like clinical testing and treatment sites.

1. How do you feel about neighborhood-level wastewater testing? [*Probes: What might be some downsides to this? How could it be helpful?*]
2. What do you think is a reasonable population size (e.g. household to a block to a building like a school to a neighborhood or city) for wastewater testing? How should the health department balance the potential benefits of more detailed health information from smaller populations with concerns for privacy?

[Twist 2 – disease] A follow up news story details how your local health department just began testing wastewater for polio and monkeypox viruses because of cases in other states. They also share a plan to test wastewater for drugs like fentanyl because of a growing number of overdose deaths in your community.

1. Does the plan to test wastewater for additional diseases sound like a good thing to you? Why or why not?
2. Who might particularly benefit from using wastewater to track community infections? Are their people or communities that might be treated unfairly because of this expanded wastewater testing?

[Twist 3 – vulnerable populations] The news story goes on to share that your local health department is collecting and testing wastewater from schools, nursing homes, and prisons because the people living or spending time at these places are more likely to get COVID or have serious illness from COVID.

1. What do you think about testing wastewater coming from these types of buildings? [*Probes: Why might this be a good approach in these types of facilities? Should the health department have any different responsibilities when testing wastewater from these populations?*]

[Twist 4 – communication/consent] A spokesperson from your local health department shares that they have been testing wastewater from high schools for drugs for the past year to provide resources to the schools with the most positive tests.

1. How do you feel about this? [*Probes: What do you see as the advantages of this approach? What are the downsides?*]
2. How should the school or health department let students, parents or teachers know about this testing? [*Probe: In what situations should the health department seek permission from people contributing to the wastewater before they start testing? In what situations might it be OK for them to proceed without explicit permission?*]

[Twist 5 – action] You learn that the school district is publishing the results of the wastewater drug testing on their website. Leadership at a high school with many positive wastewater drug tests has decided to start searching student backpacks because they worry that students are bringing and using drugs at school.

1. How do you feel about the school taking this action based on the wastewater testing results? [*Probes: Who does this help? Who might be harmed by this?* *Do you think the school needs to take action?*]
2. Are some actions OK and others not OK in response to wastewater results? For example, how would you feel about the school mandating urine drug tests for all students? Or the school offering voluntary, free, and anonymous substance use counseling for any interested student? Or the school not doing anything on an individual level but doing more education based on aggregate results? [*Probe: How would you draw the line between actions that are OK and those that overstep?*]

[Twist 6 – human DNA] The reporter interviews a concerned scientist who shares that bits of human DNA are in wastewater. This DNA could be sequenced like is done by the company 23andMe. She explains it would be hard to trace this DNA back to an individual. She wonders if the health department has thought about human genetic information contained in wastewater.

1. What concerns, if any, do you have about testing wastewater for human DNA? Can you think of some advantages of doing this?

[Twist 7 – data ownership] The news story goes on to share that your local health department has contracted with a private company to do the wastewater testing. A pharmaceutical company owns the wastewater testing company.

1. Does it matter who is testing the wastewater and owns the data? [*Probes: Who should be able to access the data? Why or why not?*]

[Twist 8 – scientific rigor] The same concerned scientist shares some limitations to wastewater testing. First, it’s hard to know who is contributing to the wastewater because people travel between communities for work and recreation and don’t consistently use the same toilets. She says it can be difficult to measure COVID-19 accurately in wastewater because the virus is present at low levels compared to testing nose swabs. Also, people excrete different amounts of the virus in their feces and some don’t excrete any virus at all. However, even with these challenges the scientist states that wastewater COVID trends usually are very similar to COVID-19 clinical test trends in a given community. She also states that wastewater testing might give a better picture of COVID infections in a community when fewer people are taking COVID tests.

1. How should your local health department balance the limitations and advantages of wastewater testing when making decisions?

Now that we have talked through several wastewater testing scenarios, I have just a few more general questions about wastewater testing.

1. Are there uses of wastewater surveillance that you think are really helpful? Are there uses that would make you uncomfortable? What are they?
2. What conditions should be put in place to make wastewater surveillance OK?
3. Is there anything else you want to share about the ethical use of public health wastewater surveillance?

Thank you for your time.
